# Challenges in the Diagnosis of Simple-Virilizing Congenital Adrenal Hyperplasia: A Case Report

**DOI:** 10.7759/cureus.29966

**Published:** 2022-10-05

**Authors:** Ritu Singh, Mukta Agarwal, Sudwita Sinha

**Affiliations:** 1 Obstetrics and Gynaecology, All India Institute of Medical Sciences, Patna, Patna, IND

**Keywords:** disorders of sexual development (dsd), ambiguous genitalia, clitoromegaly, amenorrhea, primary amenorrhea, adolescent health, hirsutism, prevention, challenges in diagnosis, congenital adrenal hyperplasia

## Abstract

Simple-virilizing congenital adrenal hyperplasia (CAH) is a rare disorder. The objective of this case report is to highlight the challenges in early diagnosis and the treatment of ambiguous genitalia so that a patient can be treated at an early stage and irreversible changes can be prevented.

A 13-year-old child, assigned female at birth, presented with the chief complaints of primary amenorrhea. The patient also reported ambiguous genitalia, male pattern hair growth, and deepening of voice (which was progressive and first noted at six years of age). She was evaluated at six years of age but not by an expert.

Ultrasound showed a normal uterus and bilateral ovaries, karyotyping XX pattern. On hormonal analysis, serum testosterone and dehydroepiandrosterone sulfate were raised but 17-hydroxyprogesterone (17-OHP) was low; this was against the diagnosis of CAH. As 17-OHP was not raised, we performed a computerized tomography scan, which showed adrenal hyperplasia. A repeat 17-OHP test showed a level of 2,000 ng/dL (>800 ng/dL is diagnostic of CAH).

We highlight several challenges in the diagnosis of the simple virilizing form of CAH. The patient’s primary complaint was primary amenorrhea, she herself did not think virilization to be important. Possibly due to social and financial issues, she had not received expert opinion in early childhood. We cannot rely solely on an investigation alone but need to see the patient as a whole. With proper and timely referral and diagnosis, we can limit serious morbidity in the form of virilization as treatments to prevent it are basic.

## Introduction

Congenital adrenal hyperplasia (CAH) is a monogenic, autosomal recessive disorder [1]. CAH is of three types, namely, salt-wasting, simple virilizing, and non-classical. The classical type includes salt-wasting and simple virilizing. The global incidence of classical CAH is 1:13,000 to 1:15,000 live births. Of these, 75% of patients have salt-wasting, and the rest have simple virilizing. Non-classical CAH is more common. Moreover, it is a common disorder in the Ashkenazi Jewish population, with one in 27 Jews affected [2]. There are various enzymes whose deficiency causes CAH, which prevents the transformation of cholesterol to cortisol. However, 21-hydroxylase (21-OH) deficiency is seen in more than 90% of CAH cases [3]. Clinical manifestations are also highly diverse depending on the levels of glucocorticoid, mineralocorticoid, and sex steroids [3].

In the late 1970s, the first neonatal screening for CAH was done in Alaska, the United States, and it became mandatory in Korea in 2006 [3,4]. Before the mandatory neonatal screening, non-fatal, simple-virilizing CAH cases remained undiagnosed and raised as men due to their cultural environment [5]. However, in India, neonatal screening for CAH is not mandatory.

Salt-wasting is associated with large gene deletions or a mutation that results in no activity of the 21-OH enzyme. In the simple-virilizing form, a point mutation results in low but detectable enzyme activity (1-2%). The fon-classical form shows compound heterozygotes, having one classic mutation and one variant allele or two variant alleles. Heterozygotes have biochemical abnormalities but typically do not have clinically significant endocrinopathy [6]. The simple-virilizing form is difficult to detect as it does not present with salt-wasting features that need admission but shows ambiguous genitalia right from birth, with virilizing features becoming evident gradually. The objective of this case presentation is to highlight the challenges in early diagnosis and the treatment of ambiguous genitalia so that patients can be treated at an early stage and the irreversible changes can be prevented.

## Case presentation

A 13-year-old patient who was assigned a female presented to the All India Institute of Medical Sciences, Patna outpatient department with the chief complaint of primary amenorrhea. She also reported ambiguous genitalia, male pattern hair growth, and deepening of voice (which was progressive and first noted at six years of age). Ambiguous genitalia was in the form of a phallus which was initially small in size but gradually increased in size. However, she was micturating normally as a female, not from the phallus. She had male pattern excessive hair growth over the upper lip, chin, chest, upper and lower abdomen, upper arm, thighs, and the upper and lower back. She had not used any hair removal methods. Her voice had changed from a female voice to a deep completely male voice.

There was no history of maternal exposure to androgens (testosterone, progesterone, aldosterone, glucocorticoids) or pesticides (dichlorodiphenyltrichloroethane, DDT), which cause endocrine disruption or virilization during pregnancy. Moreover, there was no history of hospitalization or vomiting in childhood. Regarding treatment history, the patient was evaluated only at six years of age. At that time, laboratory investigation for karyotyping was done along with an ultrasound. No treatment was advised at that time. According to the patient’s father, the doctor informed them that all that is needed for a female baby were present. There were six members in the patient’s family, four female children, our patient was the second one and all others were normal. There was no family history of similar complaints such as the male pattern of hair growth, amenorrhea, and ambiguous genitalia. There was no history of consanguinity or unexplained infant death in the family.

**Figure 1:**
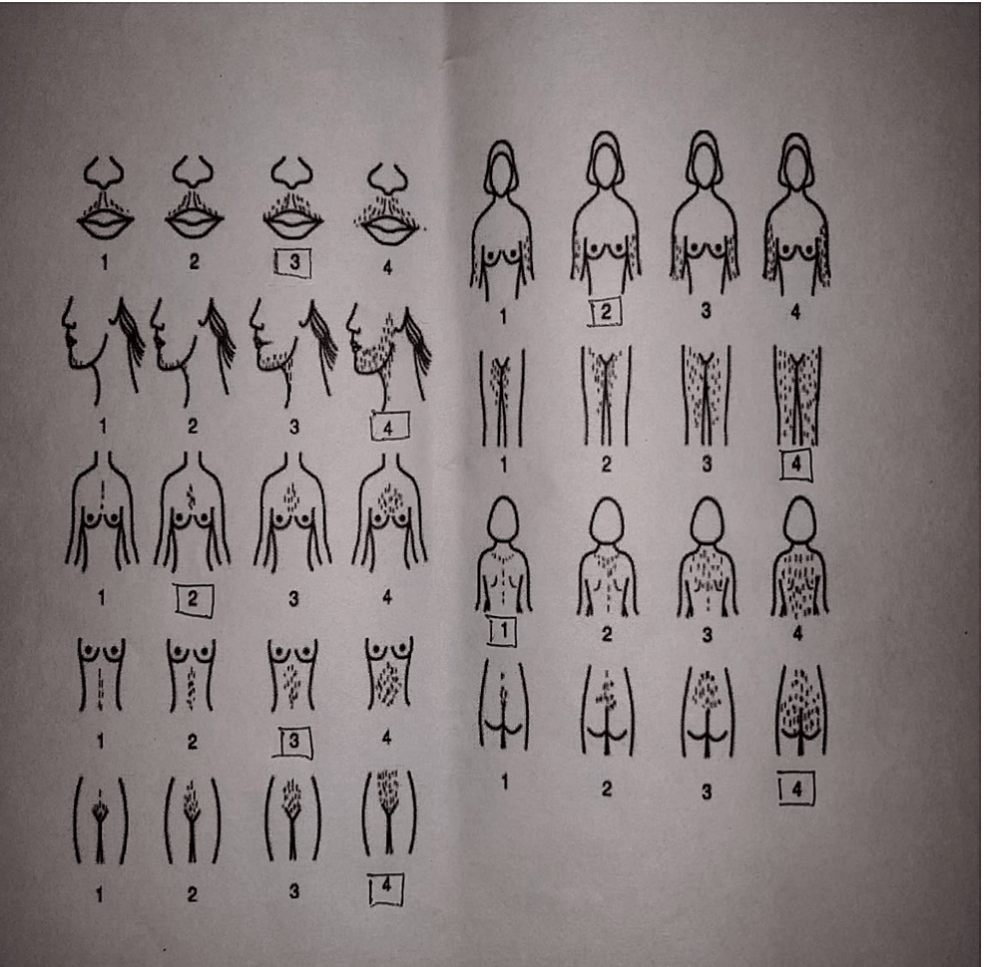
Modified Ferriman-Gallwey scoring system score of 27.

On examination, her height was 138.8 cm, which was in the 3.5th percentile for height for age according to the World Health Organization (WHO) height for age growth chart. Her weight was 42.5 kg which was in the 50.6th percentile for her age according to the WHO weight for age growth chart. On general examination, signs of virilization were present, such as acne, frontal balding, deepening of the voice, breast atrophy (Tanner stage 2), and hirsutism. According to the modified Ferriman-Gallwey scoring system, her hirsutism score was 27, which comes under the severe category (<8 is considered mild, 8-15 as moderate, and >15 as severe) (Figure 1).

**Figure 2:**
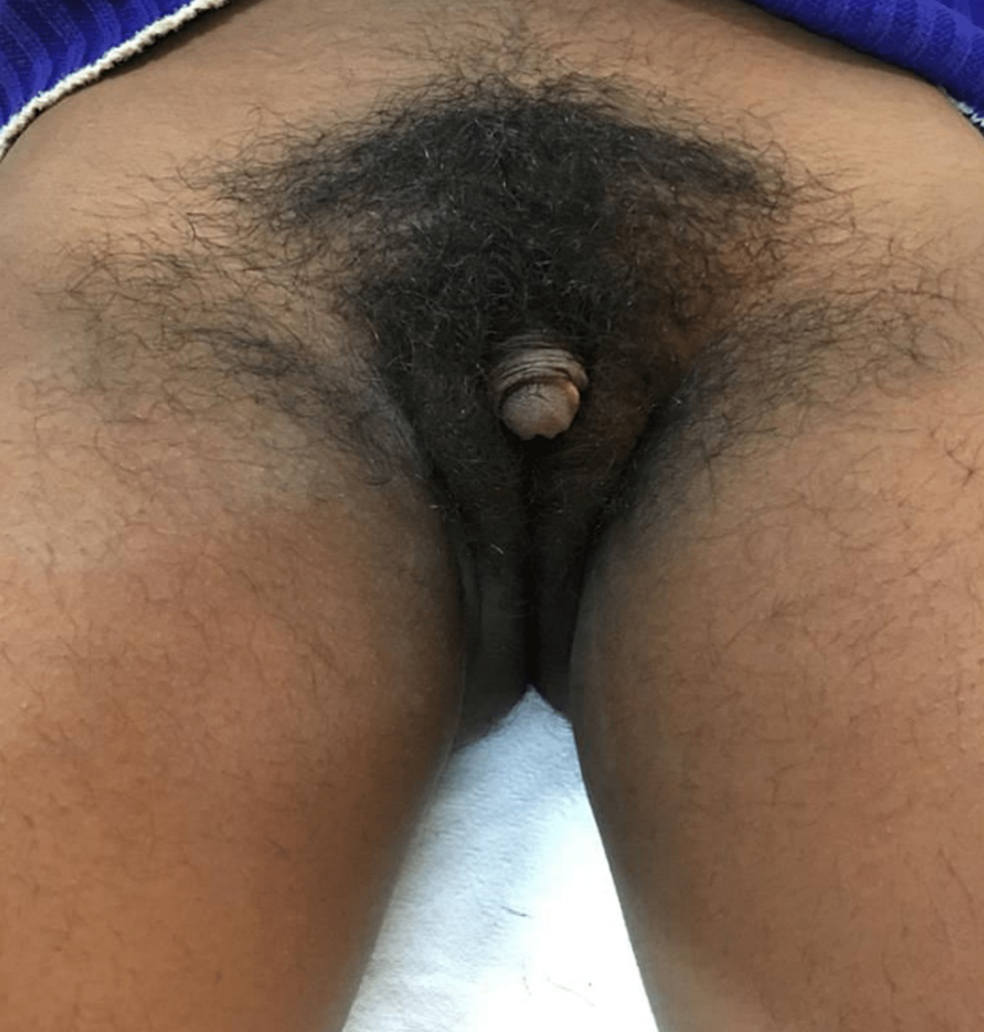
Local examination of the patient.

On conducting a systemic examination, nothing significant was found. There was no palpable mass in the inguinal region. On local examination, pubic hair was Tanner stage 5, clitoromegaly was present, the vagina was present, and the hymen was intact (Figure 2).

The length and diameter of the phallus were measured by firmly pushing the ruler against the pubic bone and pulling the phallus firmly with the other hand. The length of the phallus was 8 cm and the diameter was 2 cm. Clitoromegaly is defined as a clitoral length of more than 10 mm or clitoral index (length times width more than 35 mm). Her urethra was in a normal position just above the vagina, not at the base of the phallus which represents either an incompletely fused penile urethra (hypospadias) or a virilized urogenital sinus. Her anogenital ratio was 0.27 cm, which is the distance from the anus to the posterior fourchette of the vagina divided by the distance from the anus to the base of the clitoris (2.5 cm/9 cm = 0.27 cm). A ratio of >0.5 suggests virilization and some degree of labio-scrotal fusion. On rectal examination, the cervix was felt and the uterus was not appreciable.

**Figure 3:**
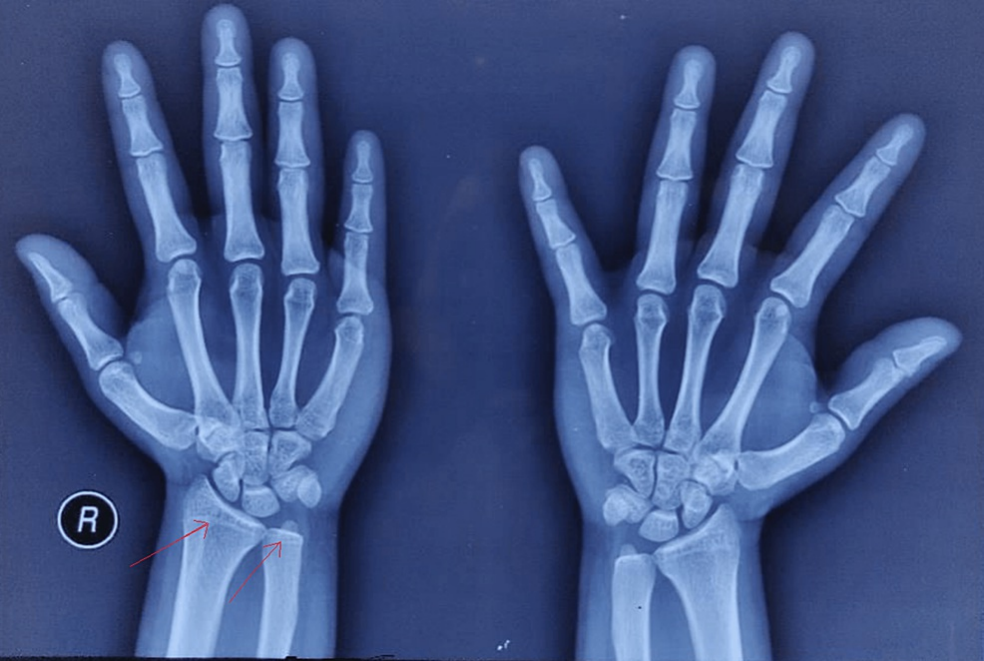
X-ray of the wrist and hand. Ossified distal epiphysis of both radius and ulna.

On further investigation, it was found that the blood investigation done at the age of six years was karyotyping, which showed an XX pattern. Genetic studies were not conducted. When she reported at 13 years of age, hormonal analysis was done, in which serum testosterone was 97.40 ng/dL (female normal: 15-70 ng/dL, level >150 ng/dL: potential androgen-producing tumor) and dehydroepiandrosterone sulfate (DHEAS) at 221 µg/dL (female normal: 40-211 µg/dL; >700 µg/dL suggests androgen-secreting adrenal tumors) was raised but 17-hydroxyprogesterone (17-OHP) at 55 ng/dL (<200 ng/dL excludes the diagnosis; >800 ng/dL is diagnostic, intermediate result require adrenocorticotropic hormone (ACTH) stimulation test) was low, which was against the diagnosis of CAH. Other investigations such as random blood sugar showed normal electrolytes. Bone age was calculated using Greulich and Pyle bone age atlas. The bone age was more than 17 years, as her distal epiphysis of both radius and ulna was ossified, which was more than her chronological age (Figure 3).

**Figure 4:**
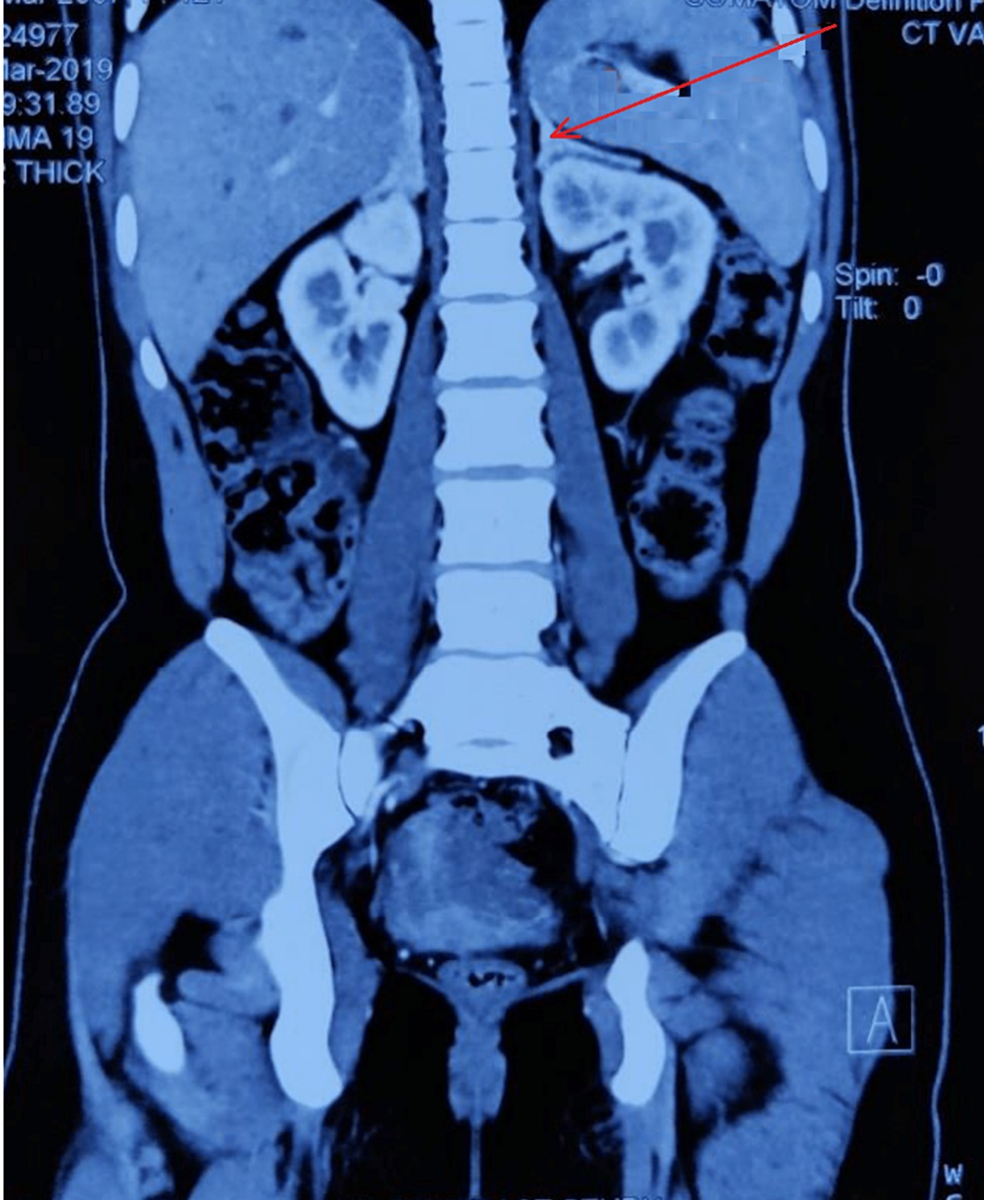
Computerized tomography scan with contrast of the abdomen showing adrenal hyperplasia (arrowhead).

As 17-OHP was not raised for diagnosis, we performed a computerized tomography (CT) scan. The CT scan showed adrenal hyperplasia (Figure 4).

Hence, a repeat 17-OHP was done which showed a level of 2,000 ng/dL (>800 ng/dL is diagnostic). The diagnosis of CAH, the simple-virilizing type, was made. To manage hirsutism, shaving, plucking, and waxing were suggested. For height, nothing could be done. We prescribed tablet hydrocortisone at a dose of 10 mg in the morning and 20 mg in the evening for three months (the recommended dose is 12-18 mg/m^2^ daily to maintain serum 17-OHP level at 400-1,200 ng/dL).

## Discussion

**Figure 5:**
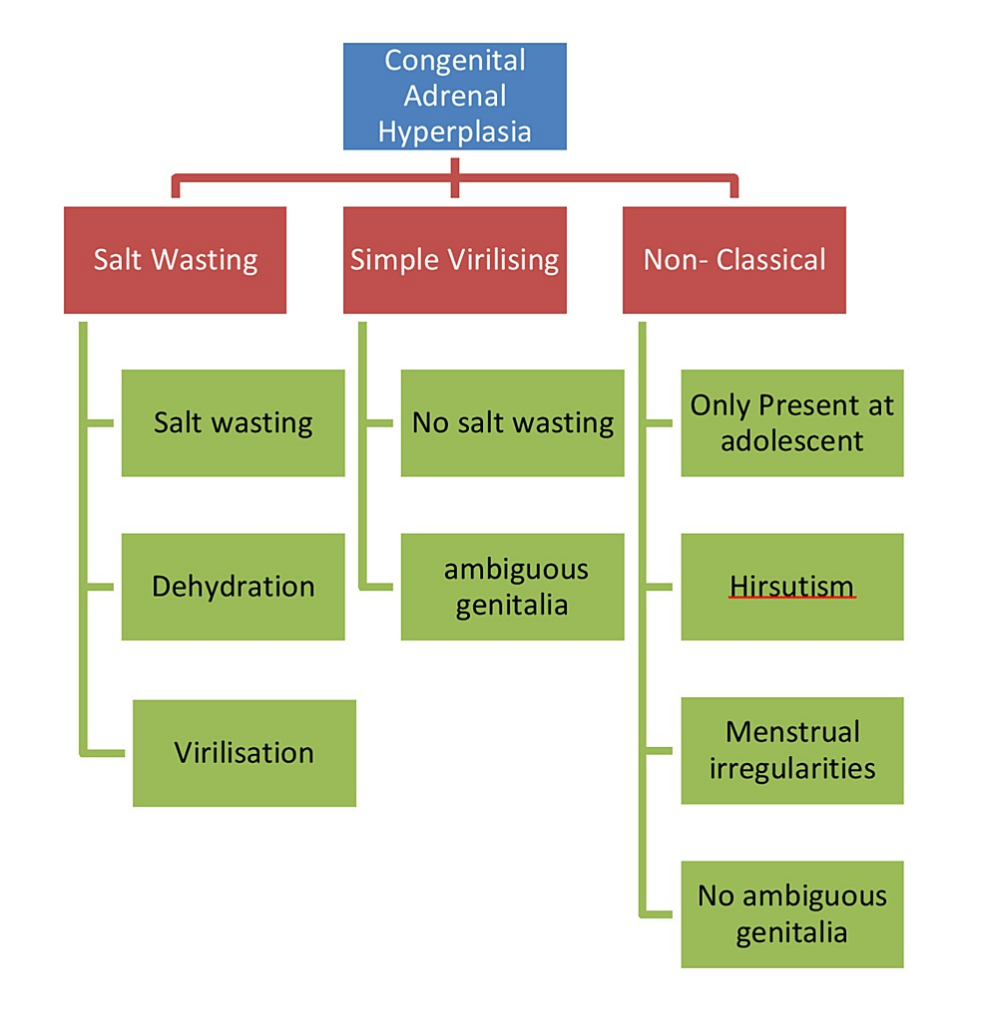
Classification of CAH with presenting complaints. CAH: congenital adrenal hyperplasia

CAH caused by 21-hydroxylase deficiency is categorized into salt-wasting, simple-virilizing, and non-classical types according to clinical manifestation (Figure 5).

Enzyme deficiency (mainly 21-OHP) is required for the synthesis of cortisol, and because of the low level of cortisol, the hypothalamus-pituitary axis reacts to it and increases ACTH secretion. It causes adrenal cortex hyperplasia, and the precursor to cortisol is mass produced and causes distinctive clinical symptoms [7]. Thus, its diagnosis was based on the increase of 17-OHP (<200 ng/dL excludes the diagnosis, >800 ng/dL is diagnostic, intermediate results require ACTH stimulation test), the metabolite of progesterone and precursor to cortisol, and DHEAS. Hormone replacement is the treatment of CAH [8].

In children, the treatment is the same for both salt-wasting and simple-virilizing forms. Glucocorticoids mainly hydrocortisone at a dose of 12-18 mg/m^2^ daily is given to promote normal growth and development. Mineralocorticoid treatment with fludrocortisone up to 0.05-0.2 mg daily can also be initiated [6].

However, the treatment of CAH in adult patients remains controversial [9]. Both in salt-wasting and simple-virilizing forms after epiphyseal closure is complete, treatment with long-acting glucocorticoids (e.g., dexamethasone, prednisolone) is preferred with bedtime dose ranging between 0.25 and 0.75 mg; hydrocortisone can also be given in single or multiple doses. However, mineralocorticoids (fludrocortisone) are given only to patients who exhibit increased plasma renin activity and aldosterone concentrations, which help in controlling 17-OHP levels [6].

According to El-Maouche et al., simple-virilizing CAH has 1-2% of 21-hydroxylase activity [3]. Therefore, serum renin and aldosterone levels were normal in the patient, preventing salt-wasting (hypotension, vomiting, diarrhea, hyponatremia, hyperkalemia, hypoglycemia, circulatory collapse) and delaying the diagnosis [3]. However, excess androgen production in utero causes virilization of external genitalia [6]. The degree of virilization can range from simple clitoromegaly to penile urethra [10].

Our patient’s history suggests it is a case of simple-virilizing CAH. Though ambiguous genitalia was noticed by her mother at six years of age, it needs to be present from birth. Her diagnosis was missed in her early childhood as she was not seen by experts at that time, which led to her short height, virilization, and male voice. We cannot revert these changes. She was so disturbed psychologically and mentally that she stopped talking as people made fun of her voice. After repeated phone calls, she did not want to come to the hospital or pursue any treatment.

As with our patients in delayed presentation cases, there should be a multidisciplinary approach and meticulous discussion with the patient. Based on the patient’s perception and preference, gender should be assigned [11]. We should try to help her through the psychosocial and legal challenges that may be faced by her. Genital reconstruction surgery should be offered if appropriate. Our patient should have undergone a thorough psychosocial assessment and counseling but this was no longer pursued due to her reluctance to attend further clinical visits.

## Conclusions

If we had intervened at an early age in our patient, we could have at least avoided her short height and advanced bone age. We cannot change her height now. She had such a deep masculine voice that she was not speaking at all at many stages of her life. Genital surgery should be avoided till puberty; however, that does not mean that nothing else should be done. We cannot rely on investigation only as a clinical sense is also necessary.

The challenges in diagnosis are (i) presentation of the patient; she presented with not having menarche yet. On asking further leading questions, she spoke of other complaints. (ii) Not having received expert opinion in early childhood. This may be due to social and financial issues her family was facing. (iii) For the first time, the 17-OHP level was low, which was against the diagnosis of CAH. Hence, with proper and timely referral and diagnosis, we can limit serious morbidity in the form of virilization as treatments to prevent it are basic.
